# Supplementary material for: Phase separation driven by interchangeable properties in the intrinsically disordered regions of protein paralogs
Source: Commun Biol. 2022 Apr 29;5:400. doi: 10.1038/s42003-022-03354-4 (PMC9054762; doi:10.1038/s42003-022-03354-4)
Supplement: Supplementary file 2 — Supplementary materials [file 42003_2022_3354_MOESM2_ESM.pdf]

Supplementary Information

Phase separation driven by interchangeable properties in the intrinsically disordered regions of protein paralogs

Shih-Hui Chiu, Wen-Lin Ho, Yung-Chen Sun, Jean-Cheng Kuo, and Jie-rong Huang

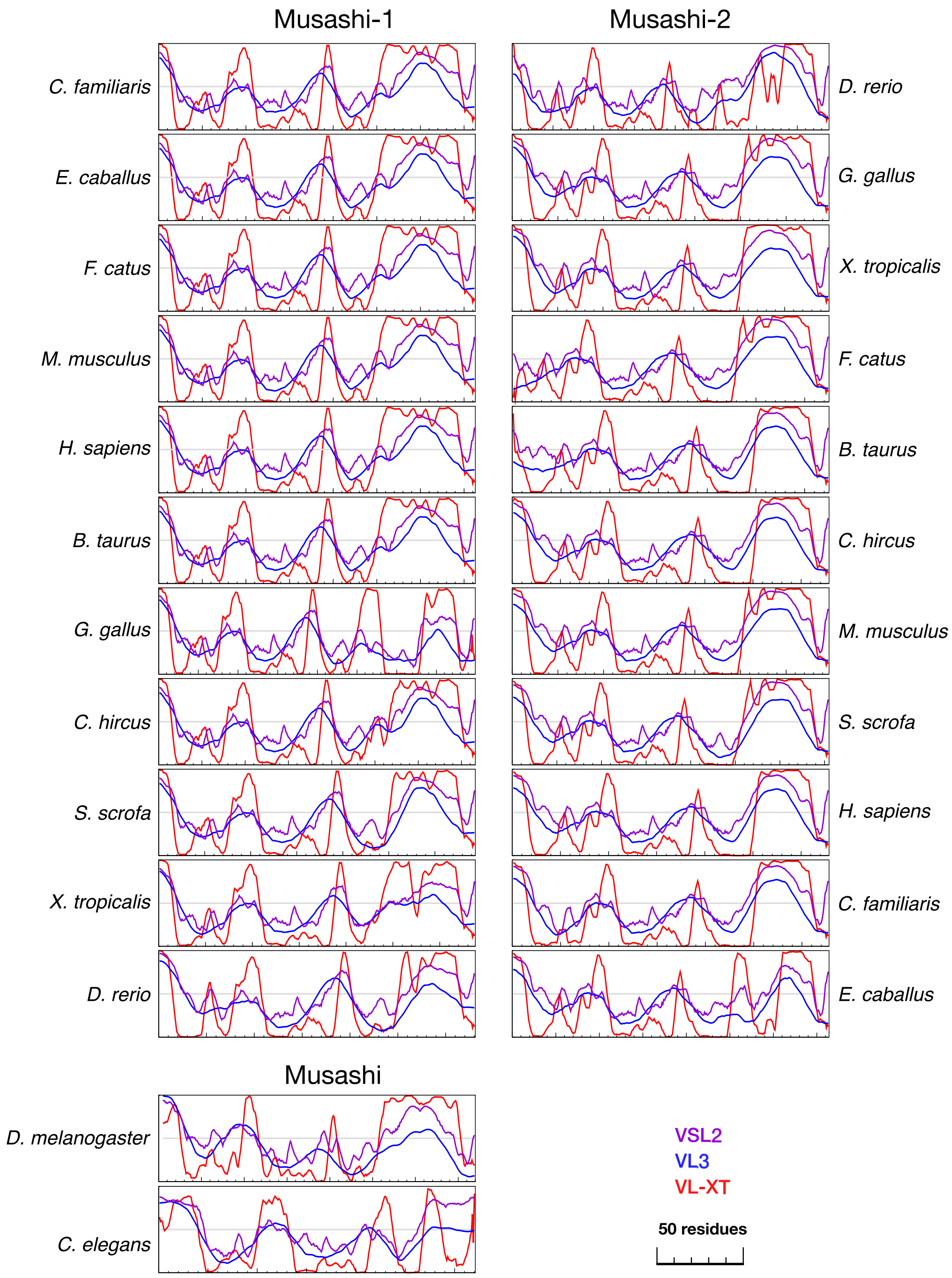

Supplementary Figure 1. Structural disorder predictions for Musashi proteins. Three algorithms: VSL2 (purple), VL3 (blue), and VL-XT (red) were used. The species are in the same order as in Fig. 1a.

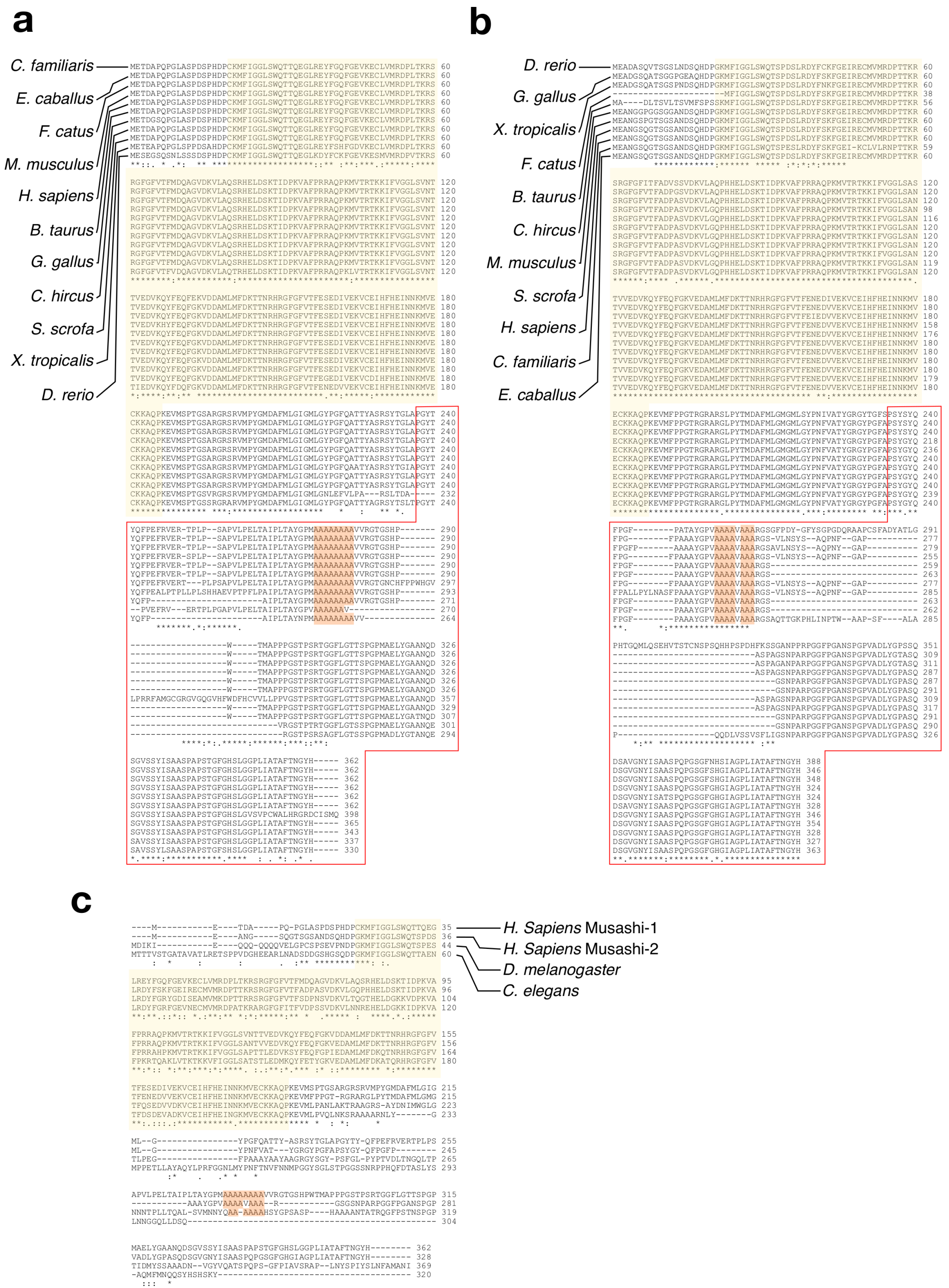

Supplementary Figure 2. Sequence alignment of Musashi proteins. a, Musashi-1 **b**, Musashi-2 in vertebrates (with species in the same order as in Fig. 1b), and **c**, human Musashi-1 and -2 with nematode and fruit fly orthologs. The RRMs are highlighted in yellow and the red boxes indicate the IDRs (according to the definition in the main text and alignment to human orthologs). The polyalanine region is highlighted in orange.

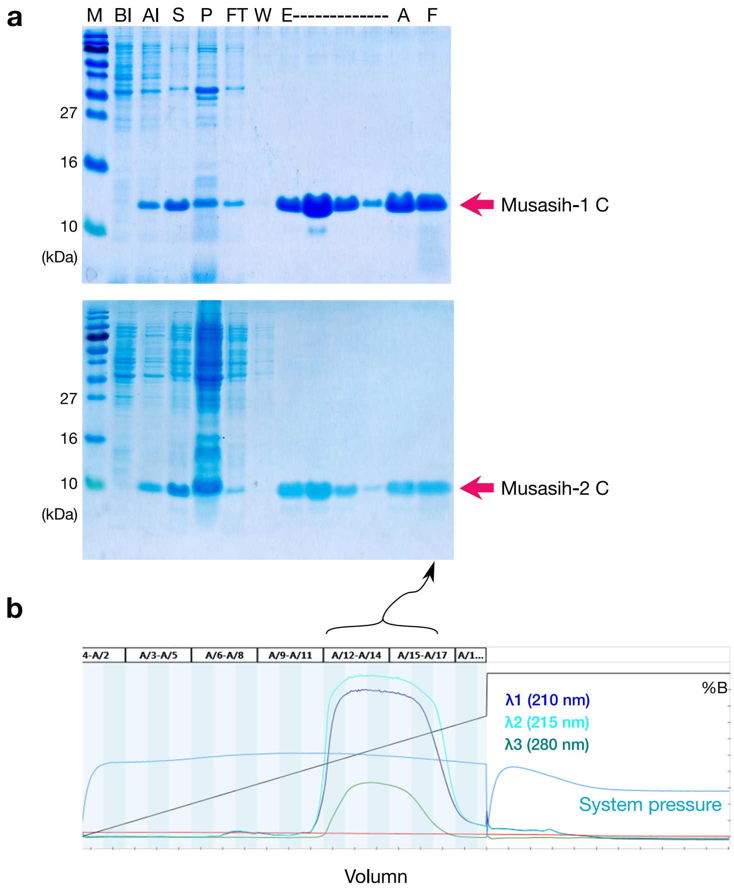

Supplementary Figure 3. Examples of protein purification results. **a**, SDS-PAGE gels of Musashi-1 (top) and Musashi-2 (bottom). M: marker; BI/AI: before/after IPTG induction; S/P: supernatant/pellet of lysed cell; FT/W/E: flow-through/wash-through/elution of the IMAC purification; A: acidified sample before loading into the C4 column; F: the final sample obtained from the HPLC column, purified and then lyophilized. **b**, A typical HPLC elution profile. The fractions collected (indicated with curly bracket) were lyophilized before use.

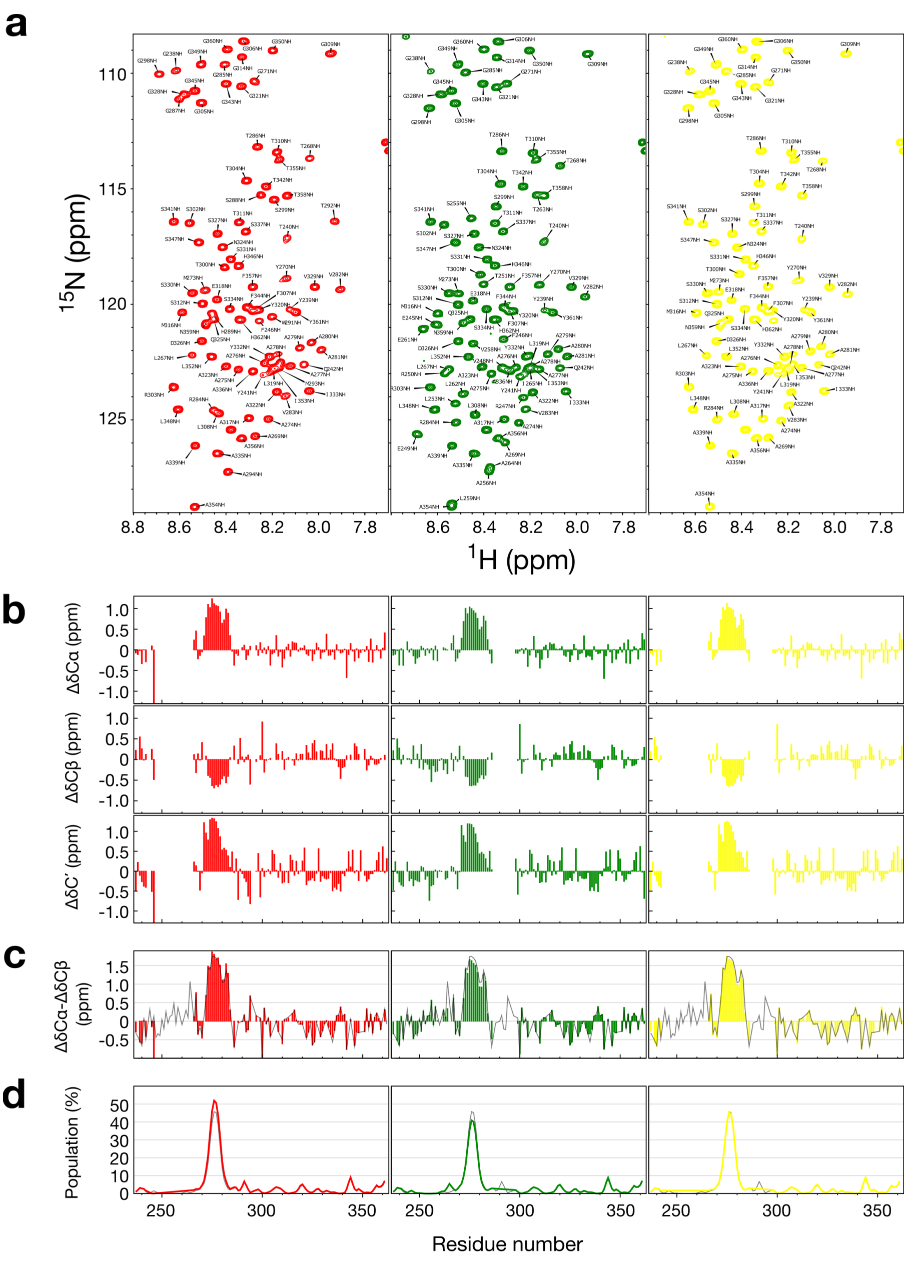

Supplementary Figure 4. NMR analysis of the Msi-1C deletion variants. a, HSQC spectra and chemical shift assignments, ΔSeq1 (red), ΔSeq2 (green), and ΔSeq1ΔSeq2 (yellow). **b**, Cα, Cβ, and C' secondary chemical shifts, **c**, secondary chemical shift differences between Cα and Cβ atoms (to eliminate chemical shift referencing errors), with the results for wild-type Msi-1C shown in gray for comparison; **d**, α-helix populations calculated with the δ2D algorithm (from H, N, Cα, Cβ, and C' chemical shifts) with the results for the wild type shown in gray. The differences between the variants and the wild type for panels (c) and (d) are shown in Fig. 3f,g in the main text.

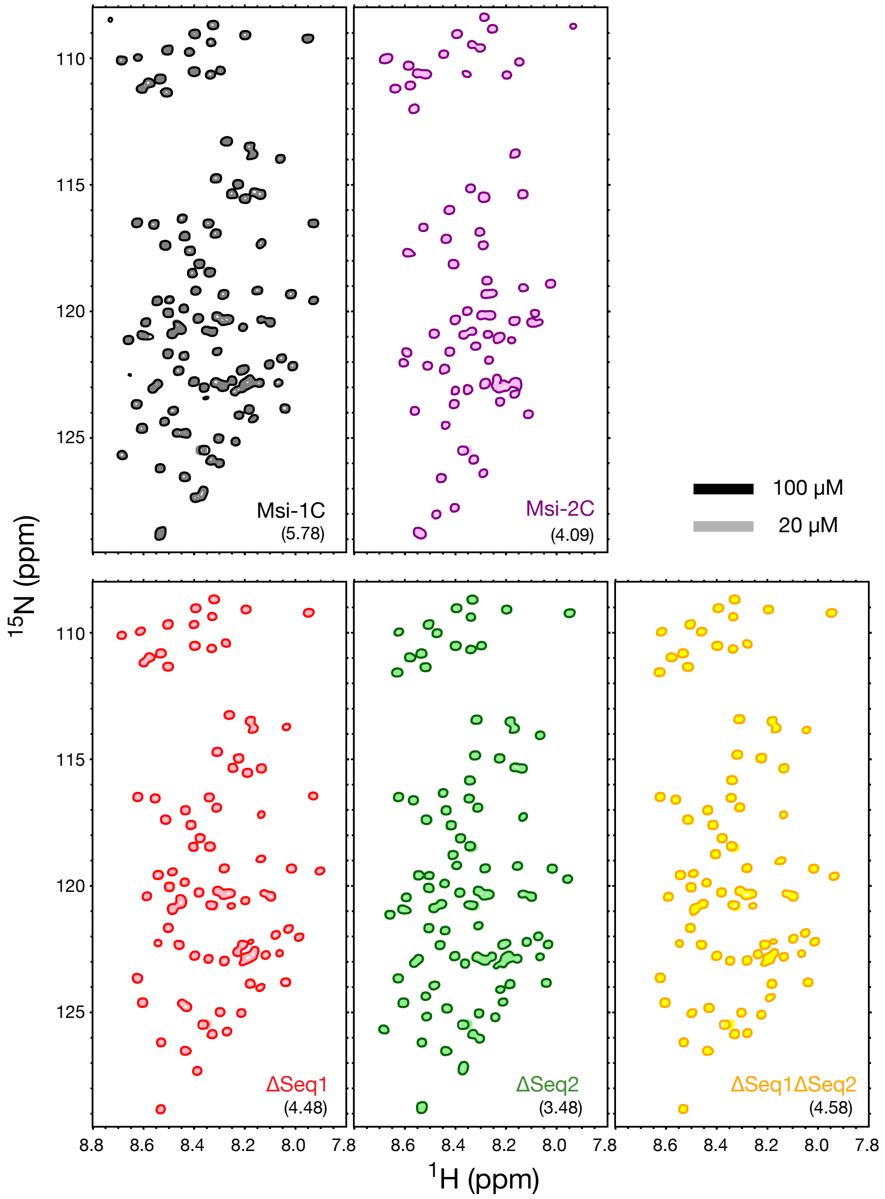

Supplementary Figure 5. The overlaid HSQC spectra of high and low concentrations samples. The HSQC spectra of the high (100 μM; open circles with dark colors) and low (20 μM; light colors) concentrations were overlaid. No significant difference is observed for all constructs. The overall intensity ratio (expected to be five) are indicated in the parathesis for each sample.

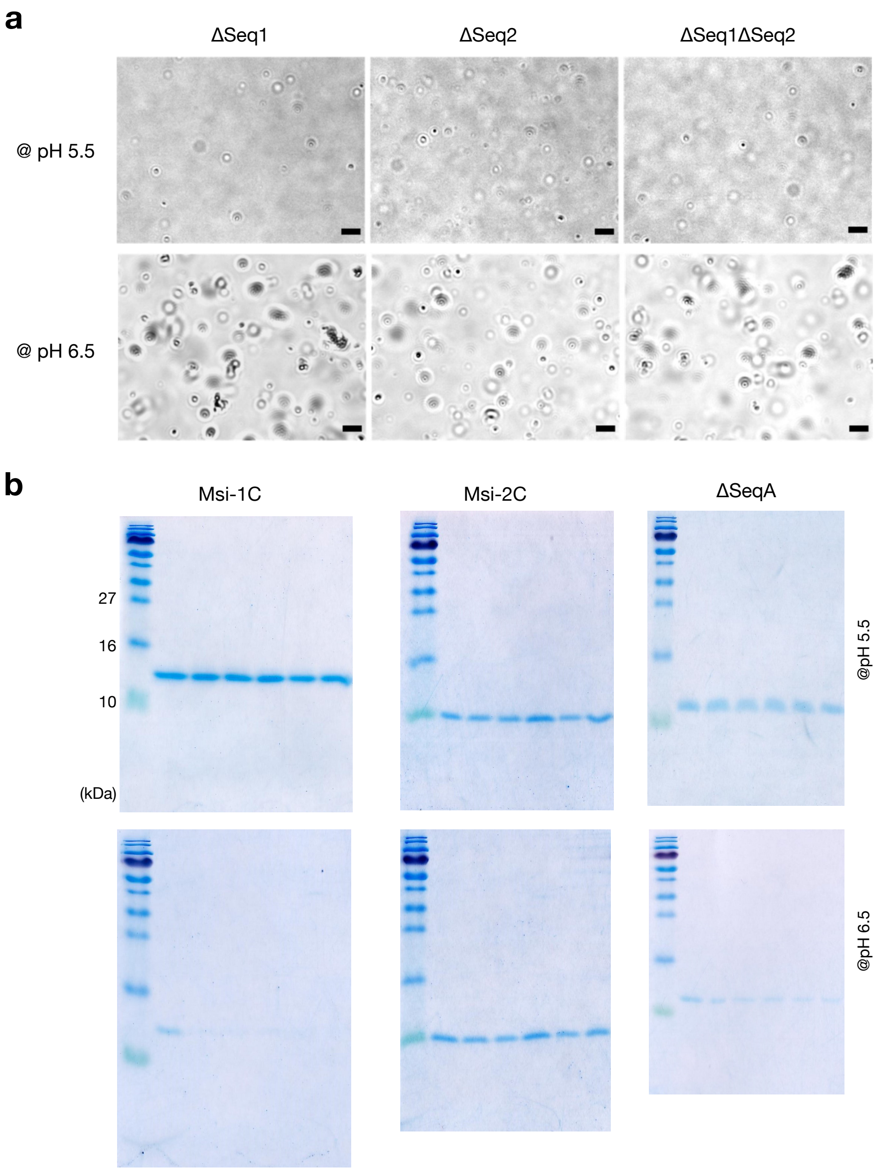

Supplementary Figure 6. Supporting images for Fig. 4. a, Optical micrographs of the condensates observed in the Msi-1C deletion constructs for ΔSeq1, ΔSeq2, and ΔSeq1ΔSeq2 at pH 5.5 and 6.5. Scale bar: 10 μm. **b**, The uncropped SDS-PAGE gels of Fig. 4f.

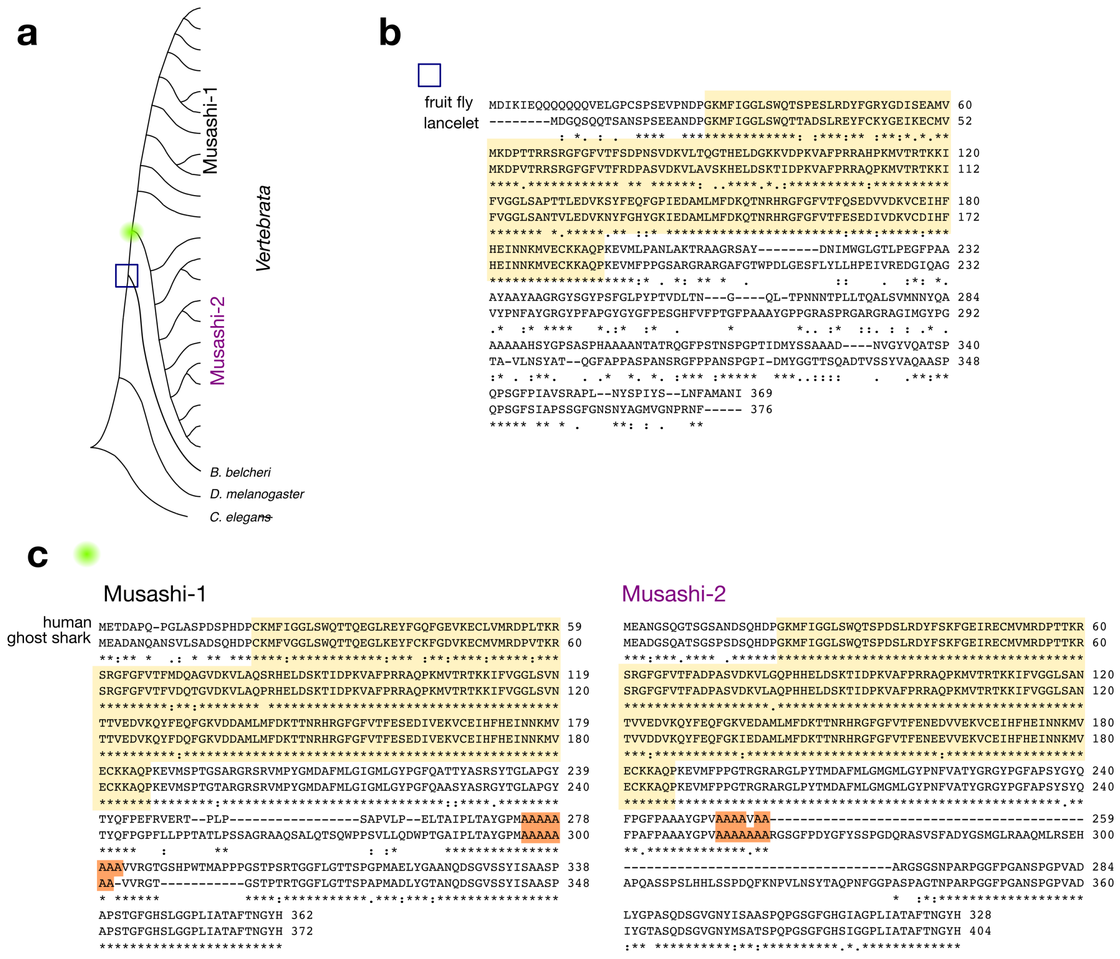

Supplementary Figure 7. Sequence analysis of primitive chordates. a, Phylogenetic tree of the Musashi family as shown in Fig. 1a with the additional lineages analyzed in this figure indicated as the blue box and green circle. **b**, Sequence alignment of fruit fly (*D. melanogaster*) and lancelet (*B. belcheri*; UniProt entry: A0A6P4YVJ9) Musashi protein. The RNA recognition motifs (as predicted by PROSITE) are indicated in yellow. **c**, Sequence alignment of human and ghost shark (*C. milii*) Musashi-1 (left, UniProt entry: V9KSD1) and Musashi-2 (right, UniProt entry: V9KVG4). The polyalanine tracts are highlighted in orange.

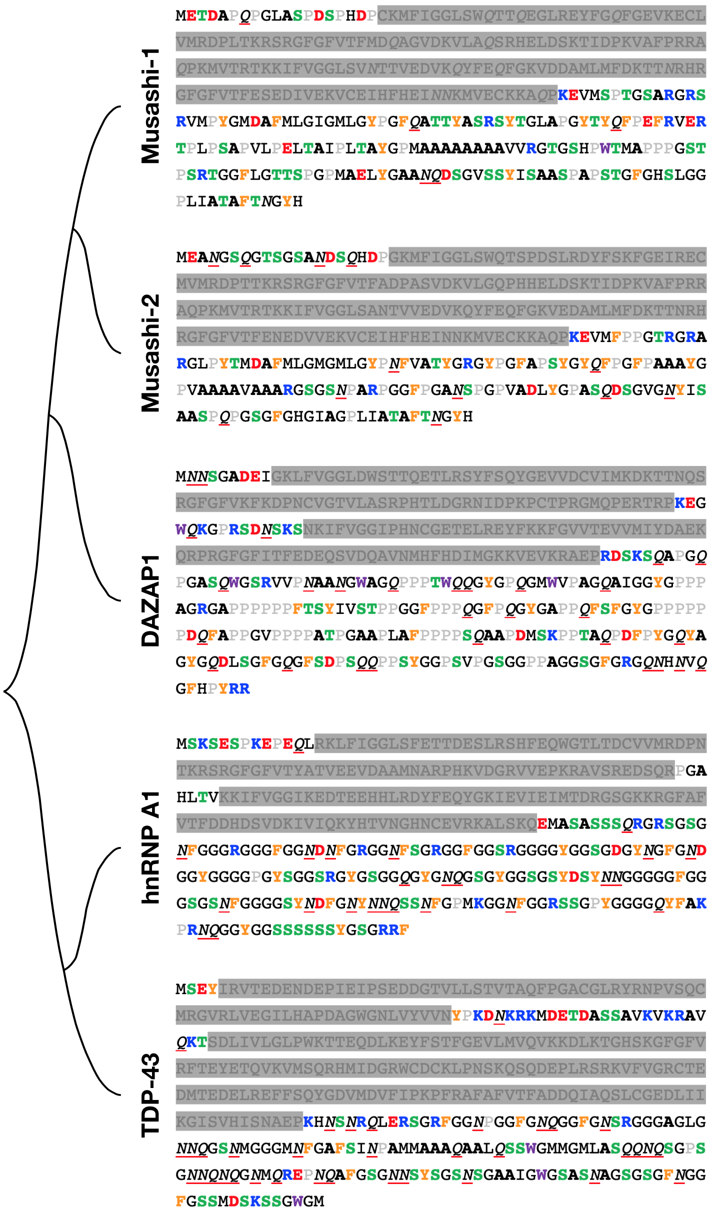

Supplementary Figure 8. Primary sequences of Musashi paralogs. The structured domains (as defined by PROSITE) are shaded in gray. The amino acids are color coded based on their physical properties (positive charge, blue; negative charge, red; F/Y, yellow; W, purple; S/T (potential phosphorylation site for the addition of negative charges), green; P, grey; A, bold black; Q/N, red underlined italic)

Supplementary Table 1. Genes used in this study.

Entry	Protein (submitted name)	Gene	Organism
O43347	Rbp Musashi homolog 1	MSI1	*Homo sapiens* (human)
Q96DH6	Rbp Musashi homolog 2	MSI2	*Homo sapiens* (human)
Q61474	Rbp Musashi homolog 1	MSI1	*Mus musculus* (mouse)
Q920Q6	Rbp Musashi homolog 2	MSI2	*Mus musculus* (mouse)
E2RK48	Musashi Rbp 1	MSI1	*Canis lupus familiaris* (dog)
A0A5F4BU43	Musashi Rbp 2	MSI2	*Canis lupus familiaris* (dog)
A0A337SLM7	Musashi Rbp 1	MSI1	*Felis catus* (cat)
A0A337SE56	Uncharacterized protein	MSI2	*Felis catus* (cat)
F6SHE7	Musashi Rbp 1	MSI1	*Equus caballus* (horse)
F7AKB0	Musashi Rbp 2	MSI2	*Equus caballus* (horse)
A2PYH9	Musashi Rbp 1	MSI1	*Bos taurus* (cattle)
A0A3Q1M610	Uncharacterized protein	MSI2	*Bos taurus* (cattle)
A0A452FXY9	Uncharacterized protein	MSI1	*Capra hircus* (goat)
A0A452EW36	Uncharacterized protein	MSI2	*Capra hircus* (goat)
I3LPG0	Musashi Rbp 1	MSI1	*Sus scrofa* (pig)
A0A287AG72	Musashi Rbp 2	MSI2	*Sus scrofa* (pig)
A0A3Q2UHP1	Uncharacterized protein	MSI1	*Gallus gallus* (chicken)
E1C1R8	Uncharacterized protein	MSI2	*Gallus gallus* (chicken)
A0A6I8QUP6	Musashi Rbp 1	MSI1	*Xenopus tropicalis* (frog)
A0A6I8SE33	Uncharacterized protein	MSI2	X *Xenopus tropicalis* (frog)
Q5BKV4	Msi1 protein	MSI1b	*Danio rerio* (zebrafish)
Q7ZW10	Msi2 protein	MSI2b	*Danio rerio* (zebrafish)
Q9VVE5	Rbp Musashi homolog Rbp6	Rbp6	*Drosophila melanogaster* (fruit fly)
G5EFS2	MuSashI (Fly neural) family	Msi-1	*Caenorhabditis elegans* (nematode)

Supplementary Table 2. Frequency and percentage of each amino acid in the RRMs and IDRs of Musashi-1 and Musashi-2.

	Msi-1 RRMs		Msi-2 RRMs		Msi-1 IDRs		Msi-2 IDRs	
aa	count	%	count	%	count	%	count	%
A	138	**5.3**	141	**5.5**	215	**15.9**	242	**19.7**
R	153	**5.9**	153	**6**	43	**3.2**	23	**1.9**
N	47	**1.8**	80	**3.1**	23	**1.7**	58	**4.7**
D	157	**6.1**	151	**5.9**	12	**0.9**	27	**2.2**
C	44	**1.7**	34	**1.3**	5	**0.4**	2	**0.2**
Q	117	**4.5**	82	**3.2**	23	**1.7**	45	**3.7**
E	165	**6.4**	153	**6**	38	**2.8**	1	**0.1**
G	238	**9.2**	253	**9.9**	172	**12.7**	200	**16.3**
H	57	**2.2**	64	**2.5**	36	**2.7**	28	**2.3**
I	78	**3.0**	70	**2.7**	32	**2.4**	35	**2.8**
L	132	**5.1**	104	**4.1**	88	**6.5**	36	**2.9**
K	189	**7.3**	187	**7.3**	0	**0**	2	**0.2**
M	142	**5.5**	141	**5.5**	31	**2.3**	1	**0.1**
F	187	**7.2**	209	**8.2**	55	**4.1**	65	**5.3**
P	139	**5.4**	155	**6.1**	184	**13.6**	169	**13.8**
S	148	**5.7**	128	**5**	132	**9.8**	133	**10.8**
T	185	**7.1**	165	**6.5**	130	**9.6**	33	**2.7**
W	11	**0.4**	11	**0.4**	11	**0.8**	1	**0.1**
Y	63	**2.4**	66	**2.6**	63	**4.7**	76	**6.2**
V	202	**7.8**	200	**7.9**	60	**4.4**	52	**4.2**

Supplementary Table 3. Primers used in this study.

Constructs	Primers (5'–3')
MSI-1[237-362]	*Fw*-GGAGATATACATATGCCTGGCTACACCTAC<br>*Rv*-GTAGGTGTAGCCAGGCATATGTATATCTCCTTCTTAAAGTT
MSI-1-ΔSeq1	Fw-GAATTCCCTCTCACTGCCTAC<br>*Rv*-GAGAGGGAATTCGGGGAACTGG
MSI-1-ΔSeq2	*Fw*-GGGACAGGTTCGACTCCCAG<br>*Rv*-CGAACCTGTCCCTCGAACCAC
MSI-1-ΔSeq1Seq2	The same as ΔSeq1 and ΔSeq2
MSI-1-ΔSeqA	*Fw*-GCCATTGGCTCTCACCCCTGG<br>*Rv*-AGAGCCAATGGCTGTAAGCTC
MSI-2[235-328]	*Fw*-CCAAGCTATGGCTATCAG<br>*Rv*-ATAGCCATAGCTTGGCATATGTATATCTCCTTCTTAAAGTT
