# Supplementary material for: Phase separation driven by interchangeable properties in the intrinsically disordered regions of protein paralogs
Source: Commun Biol. 2022 Apr 29;5:400. doi: 10.1038/s42003-022-03354-4 (PMC9054762; doi:10.1038/s42003-022-03354-4)
Supplement: Supplementary file 3 — Description of Additional Supplementary Files [file 42003_2022_3354_MOESM3_ESM.pdf]

Description of Additional Supplementary Files

File name: Supplementary Data 1
Description: 341 RBPs in one of the paralog families in the OrthoMCL database.

File name: Supplementary Data 2
Description: 749 RBPs in one of the paralog families based on sequence similarity.

File name: Supplementary Data 3
Description: Source data underlying Figs. 2a, 3c, 4a, 4e, 4f.
