# Supplementary material for: Phase separation driven by interchangeable properties in the intrinsically disordered regions of protein paralogs
Source: Commun Biol. 2022 Apr 29;5:400. doi: 10.1038/s42003-022-03354-4 (PMC9054762; doi:10.1038/s42003-022-03354-4)
Supplement: Supplementary file 7 — Reporting Summary [file 42003_2022_3354_MOESM7_ESM.pdf]

nature portfolio

Corresponding author(s): Jie-rong Huang

Last updated by author(s): Apr 8, 2022

Reporting Summary

Nature Portfolio wishes to improve the reproducibility of the work that we publish. This form provides structure for consistency and transparency in reporting. For further information on Nature Portfolio policies, see our Editorial Policies and the Editorial Policy Checklist.

Statistics

For all statistical analyses, confirm that the following items are present in the figure legend, table legend, main text, or Methods section.

n/a	Confirmed	
☐	☒	The exact sample size (n) for each experimental group/condition, given as a discrete number and unit of measurement
☐	☒	A statement on whether measurements were taken from distinct samples or whether the same sample was measured repeatedly
☒	☐	The statistical test(s) used AND whether they are one- or two-sided<br>*Only common tests should be described solely by name; describe more complex techniques in the Methods section.*
☒	☐	A description of all covariates tested
☐	☒	A description of any assumptions or corrections, such as tests of normality and adjustment for multiple comparisons
☐	☒	A full description of the statistical parameters including central tendency (e.g. means) or other basic estimates (e.g. regression coefficient) AND variation (e.g. standard deviation) or associated estimates of uncertainty (e.g. confidence intervals)
☒	☐	For null hypothesis testing, the test statistic (e.g. F, t, r) with confidence intervals, effect sizes, degrees of freedom and P value noted<br>*Give P values as exact values whenever suitable.*
☒	☐	For Bayesian analysis, information on the choice of priors and Markov chain Monte Carlo settings
☒	☐	For hierarchical and complex designs, identification of the appropriate level for tests and full reporting of outcomes
☒	☐	Estimates of effect sizes (e.g. Cohen's d, Pearson's r), indicating how they were calculated

Our web collection on statistics for biologists contains articles on many of the points above.

Software and code

Policy information about availability of computer code

Data collection	Bruker TopSpin 3.0; Bruker TopSpin 2.0;
Data analysis	NMRPipe 8.1; Sparky 3; PONDR; PLAAC; BLAST; MAGA X

For manuscripts utilizing custom algorithms or software that are central to the research but not yet described in published literature, software must be made available to editors and reviewers. We strongly encourage code deposition in a community repository (e.g. GitHub). See the Nature Portfolio guidelines for submitting code & software for further information.

Data

Policy information about availability of data

All manuscripts must include a data availability statement. This statement should provide the following information, where applicable:

- Accession codes, unique identifiers, or web links for publicly available datasets
- A description of any restrictions on data availability
- For clinical datasets or third party data, please ensure that the statement adheres to our policy

The source data underlying Figs. 2a, 3c, 4a, 4e, 4f, are provided as a Source Data file. The chemical shift assignments for generating Figs. 2b, 2c, 2d, 3d, 3e, 3f, 3g are deposited in the Biological Magnetic Resonance Bank (BMRB): 51204, 51205, 51206, 51207, 51208. Supplementary Tables 1 and 2 contain the gene names or data for reproducing Figs. 1a, 1b, 1c, 1d, 2e, 3a, 6a, 6b, 6c, 6e, 6f. Scripts for reproducing Fig. 5 are deposited in http://github.com/allmwh/helix_score

March 2021

Field-specific reporting

Please select the one below that is the best fit for your research. If you are not sure, read the appropriate sections before making your selection.

☒ Life sciences ☐ Behavioural & social sciences ☐ Ecological, evolutionary & environmental sciences

For a reference copy of the document with all sections, see nature.com/documents/nr-reporting-summary-flat.pdf

Life sciences study design

All studies must disclose on these points even when the disclosure is negative.

Sample size	All observation of this purified protein (in vitro study only in this work) were repeated at least three times.
Data exclusions	No data were excluded.
Replication	The turbidity, micrograph, and FRAP studies were repeated at least three times on independently prepared samples. All attempts at replication on NMR experiments were successful (at least once).
Randomization	No sample selection was carried out, samples were chosen independently and randomly
Blinding	Not applicable

Reporting for specific materials, systems and methods

We require information from authors about some types of materials, experimental systems and methods used in many studies. Here, indicate whether each material, system or method listed is relevant to your study. If you are not sure if a list item applies to your research, read the appropriate section before selecting a response.

Materials & experimental systems

n/a	Involved in the study
☒	☐ Antibodies
☒	☐ Eukaryotic cell lines
☒	☐ Palaeontology and archaeology
☒	☐ Animals and other organisms
☒	☐ Human research participants
☒	☐ Clinical data
☒	☐ Dual use research of concern

Methods

n/a	Involved in the study
☒	☐ ChIP-seq
☒	☐ Flow cytometry
☒	☐ MRI-based neuroimaging
